# A rare presentation of a solitary fibrous tumour

**DOI:** 10.1007/s12471-023-01775-2

**Published:** 2023-04-05

**Authors:** Gonçalo N. Ferraz Costa, Fátima Franco, Rogério P. C. Teixeira

**Affiliations:** 1 Serviço de Cardiologia, Centro Hospitalar e Universitário de Coimbra, Coimbra, Portugal; 2 Faculdade de Medicina, Universidade de Coimbra, Coimbra, Portugal; 3 Coimbra Institute for Clinical and Biomedical Research, Coimbra, Portugal

A 67-year-old woman was referred to our centre because of an intracardiac mass. Her medical history included a retroperitoneal tumour (2015), lung metastases (2020) and bilateral breast implantation. Transthoracic echocardiography had a poor acoustic window due to the breast implants. Transoesophageal echocardiography showed a mass in the left atrium (6.2 × 4.4 cm) that occluded most of the atrium, had an uneven shape and showed heterogeneous echogenicity. Due to its size, the mass, which seemed to derive from the left pulmonary vein, restricted transmitral flow; mean transmitral gradient was 7 mm Hg (Fig. 1a, b, and see Videos 1 and 2 in Electronic Supplementary Material). Cardiac computed tomography (CT) angiography showed a vascularised cardiac mass with reduced mobility and uneven contrast enhancement; there was no cardiac infiltration. CT showed the tumour entered the heart from the left upper pulmonary vein and revealed left superior pulmonary vein thrombosis (Fig. 1c, d, and see Videos 3–5 in Electronic Supplementary Material); lung window study indicated several lung metastases (Fig. 1e). Transthoracic biopsy of one metastasis and subsequent pathological examination revealed a solitary fibrous tumour (Fig. 1f). Despite chemotherapy, the patient died. This report is an example of a slow-growing, solitary fibrous tumour turning malignant [1, 2].

**Fig. 1:**
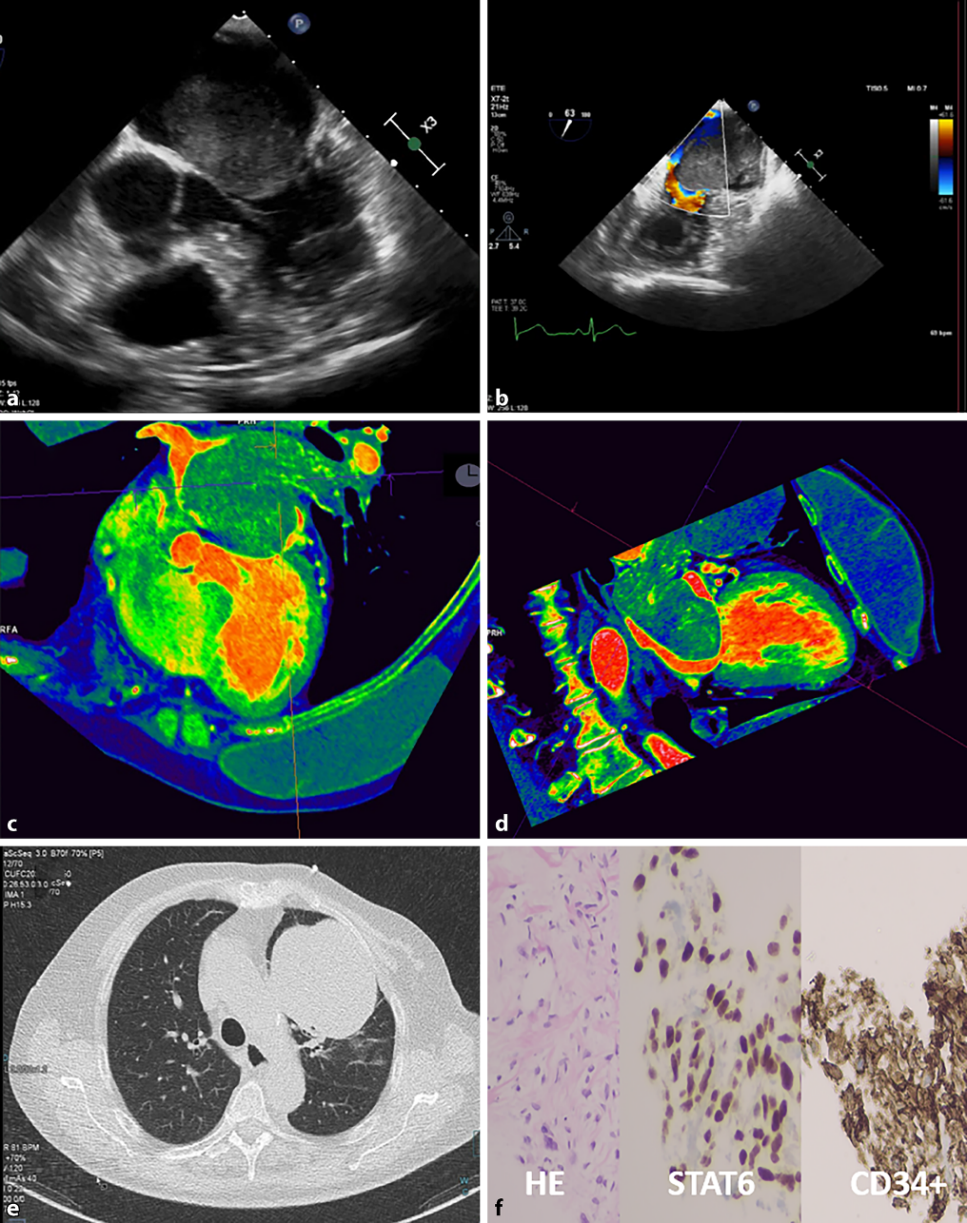
Assessment of solitary fibrous tumour. **a**, **b** Transoesophageal echocardiograms. **c**, **d** Cardiac computed tomography (CT) scan showing vascularised cardiac mass in left atrium, with reduced mobility and heterogeneous contrast enhancement. This mass entered the heart from the left upper pulmonary vein; left superior pulmonary vein thrombosis was also seen on CT. **e** CT scan with lung window showing multiple pulmonary nodules in both pulmonary camps. **f** Pathological examination with haematoxylin & eosin (HE), STAT6 nuclear and CD34 cytoplasmic stainings showing nuclear atypia of fusiform cells

## Supplementary Information

**Video 1** Transoesophageal echocardiogram showing mass (size: 6.2 × 4.4 cm) that fills large part of left atrial volume, with irregular contour and heterogeneous echogenicity. This mass originated from left superior pulmonary vein and was suggestive of malignancy

**Video 2** Transoesophageal echocardiogram showing left atrial mass that causes restrictive transmitral flow with transmitral maximal/mean gradients of 14/7 mm Hg, which indicated functional mitral stenosis

**Video 3** Cardiac computed tomography angiogram with retrospective gating and multiphasic reconstruction in axial plane showing vascularised, low-density tumour mass (size: 6.5 × 4.3 cm; 60–70 HU) that occupies three-quarters of left atrial volume. This mass expanded to left atrial appendage

**Video 4** Colour cardiac computed tomography angiogram with retrospective gating and multiphasic reconstruction showing vascularised mass in left atrium, with reduced mobility and heterogeneous contrast enhancement. No invasion of valves, left ventricle, right chambers, coronary arteries or aorta

**Video 5 **Colour cardiac computed tomography angiogram with retrospective gating and multiphasic reconstruction showing left atrial mass entering heart from left upper pulmonary vein, with associated left superior pulmonary vein thrombosis
